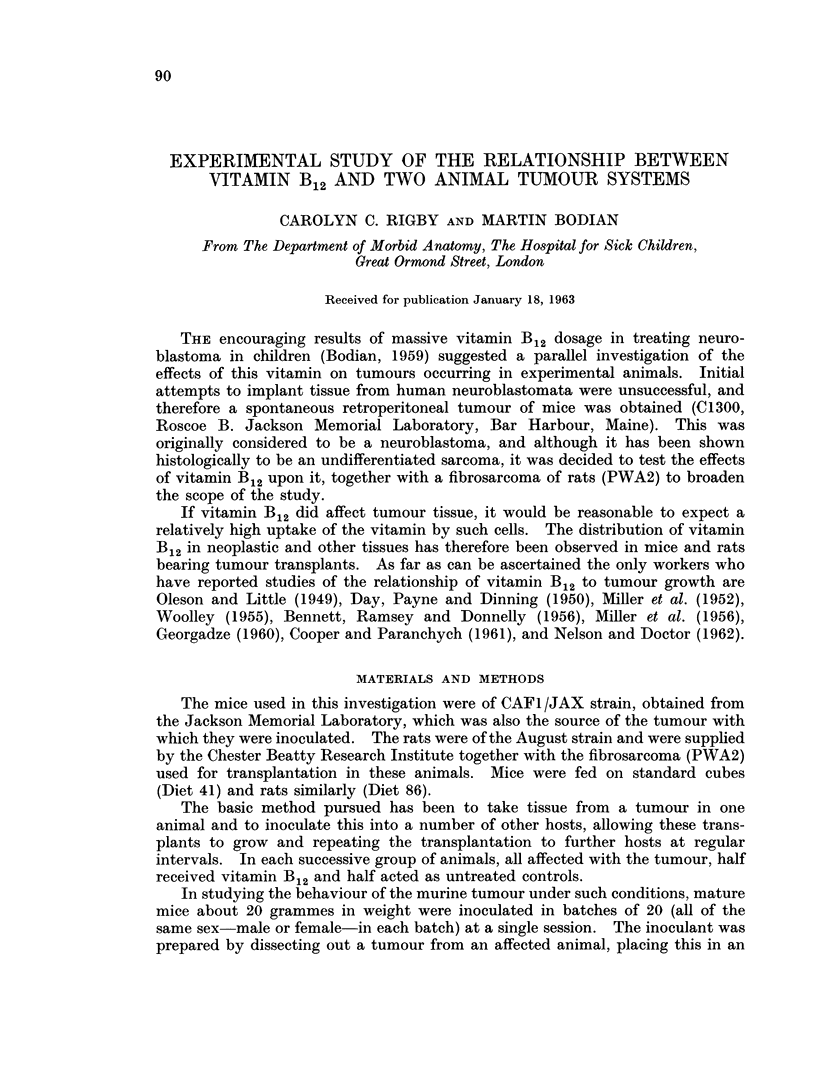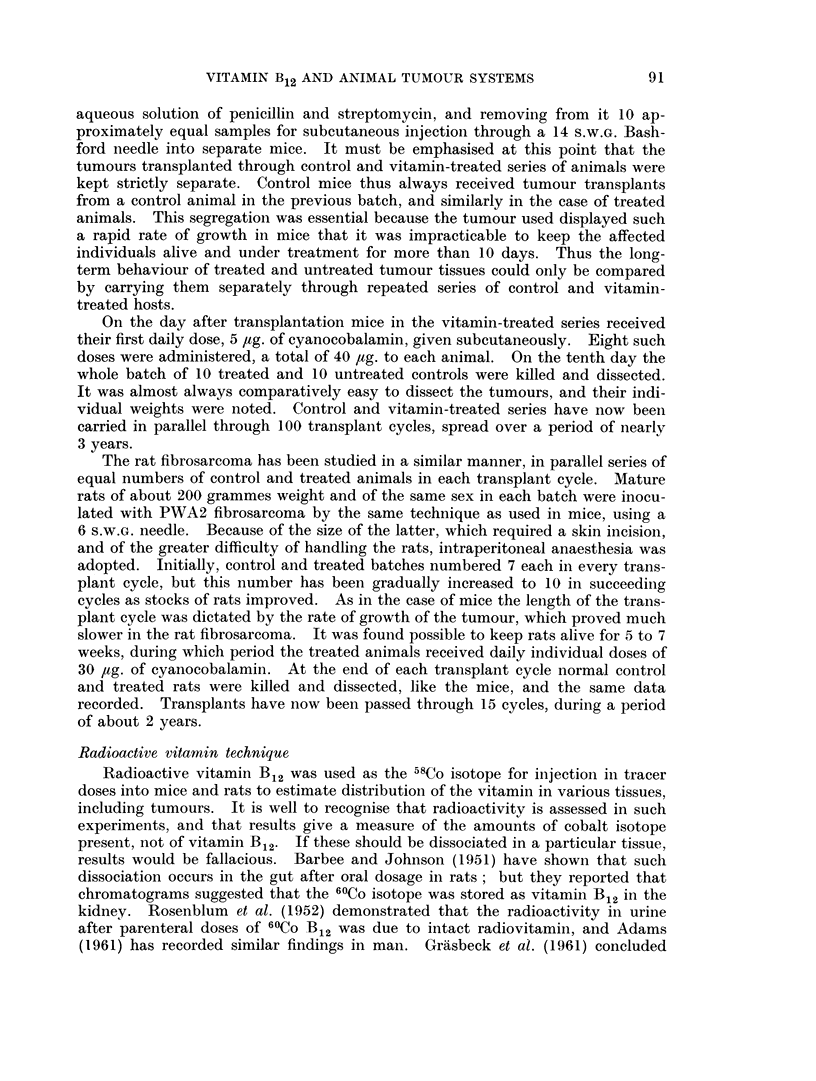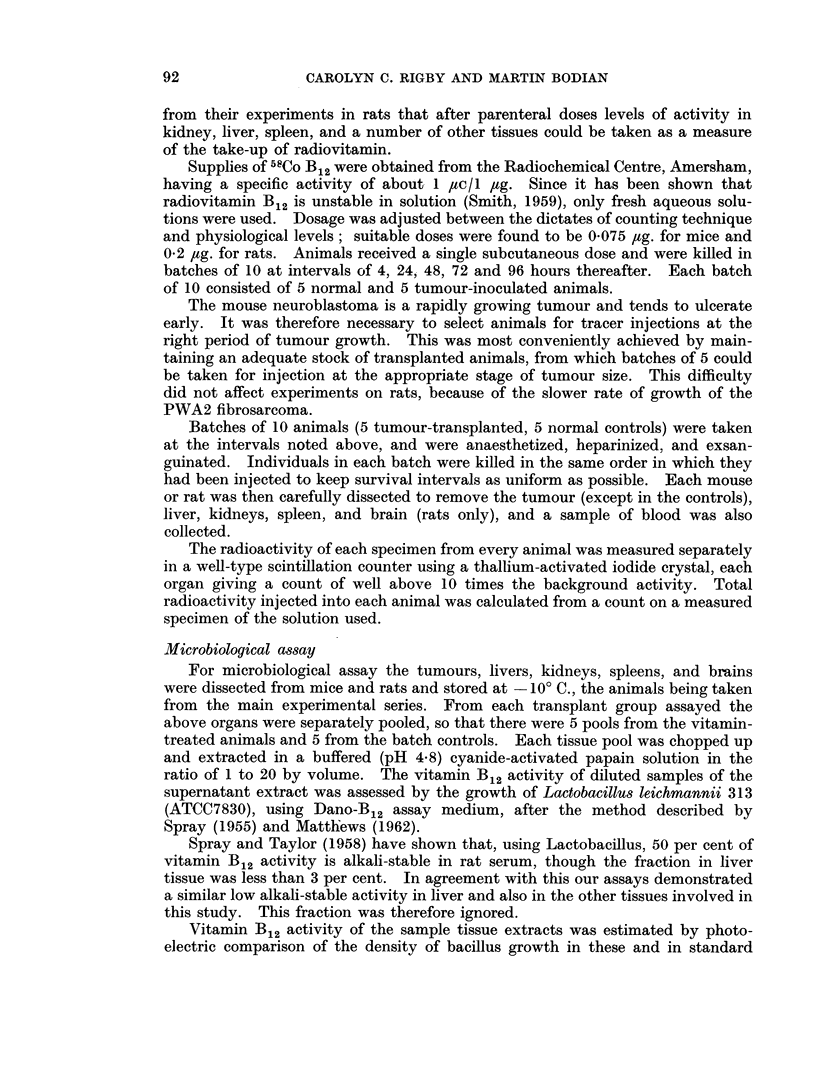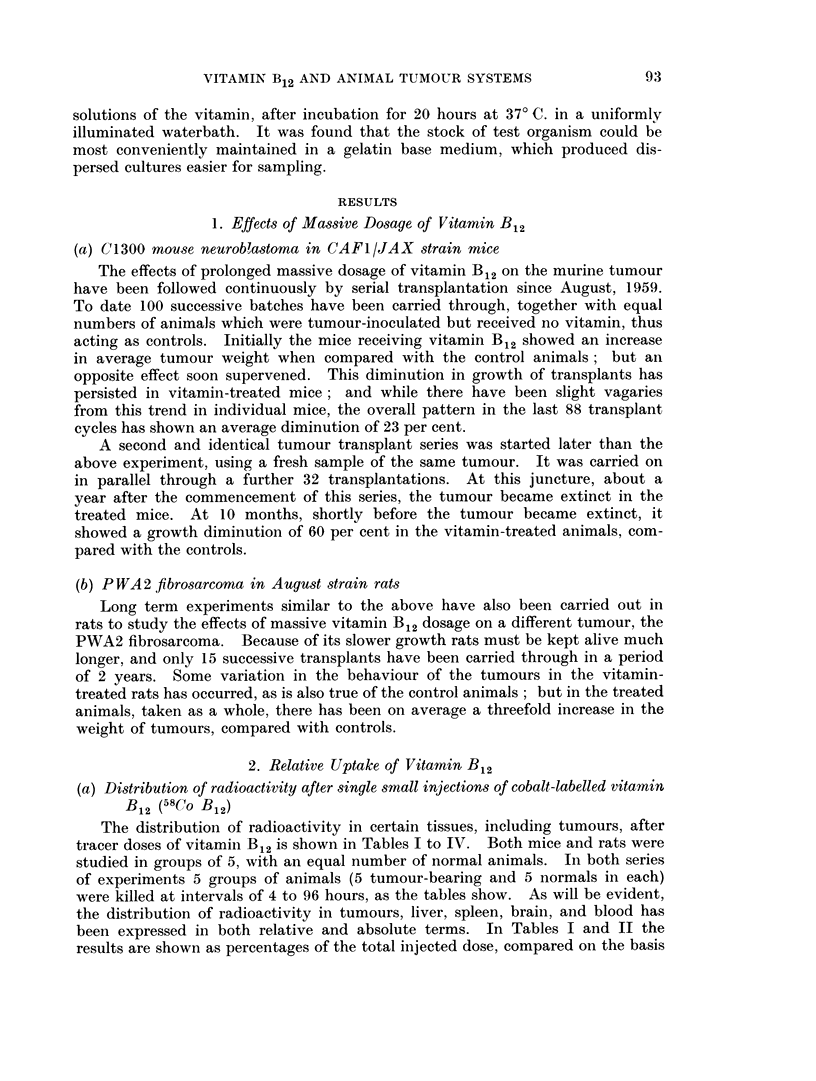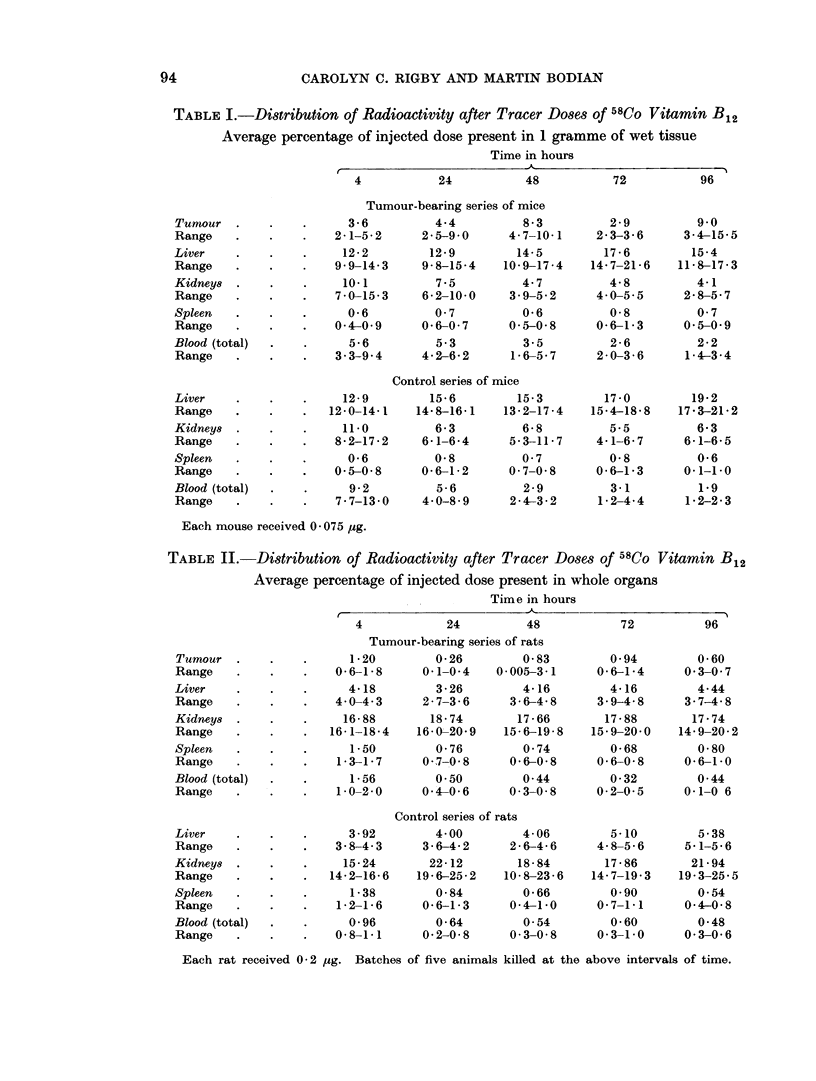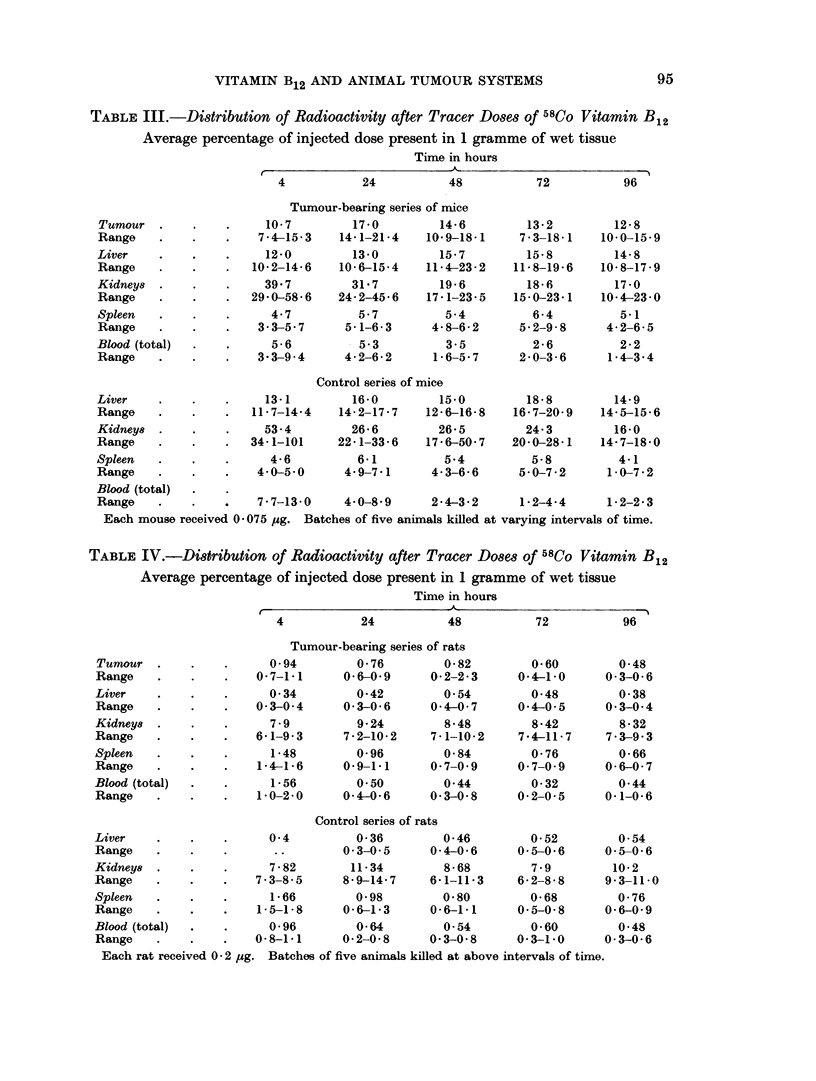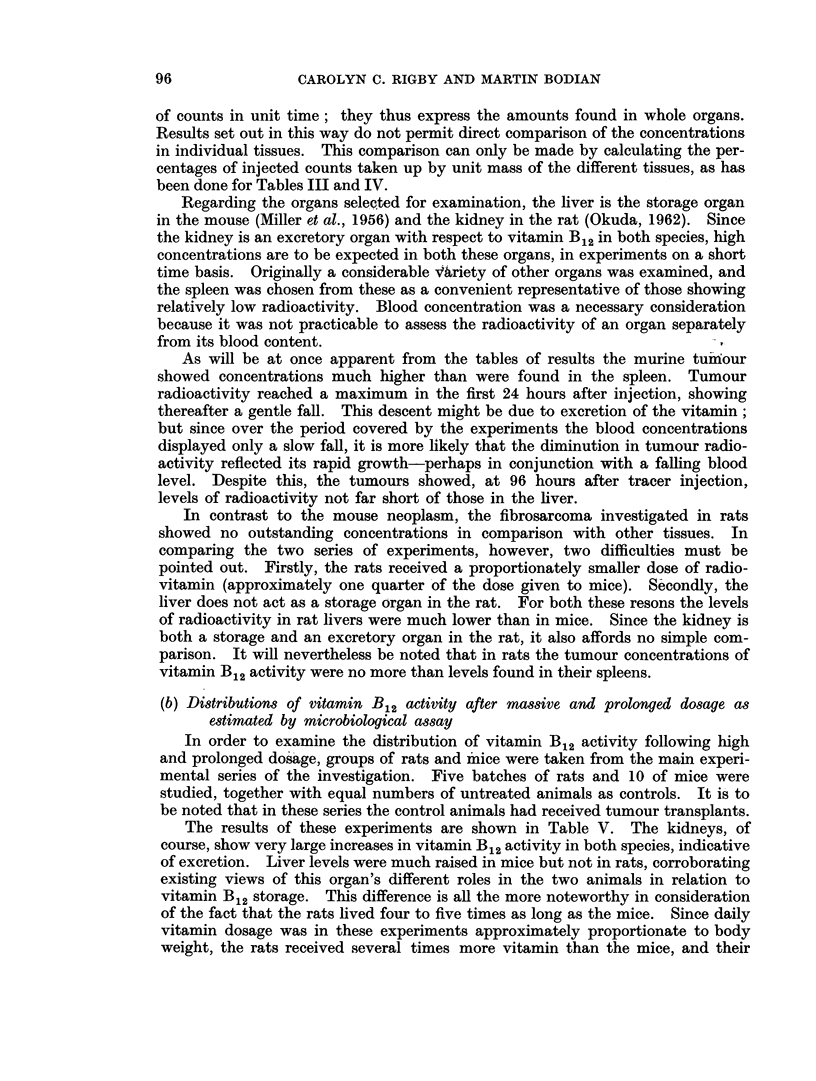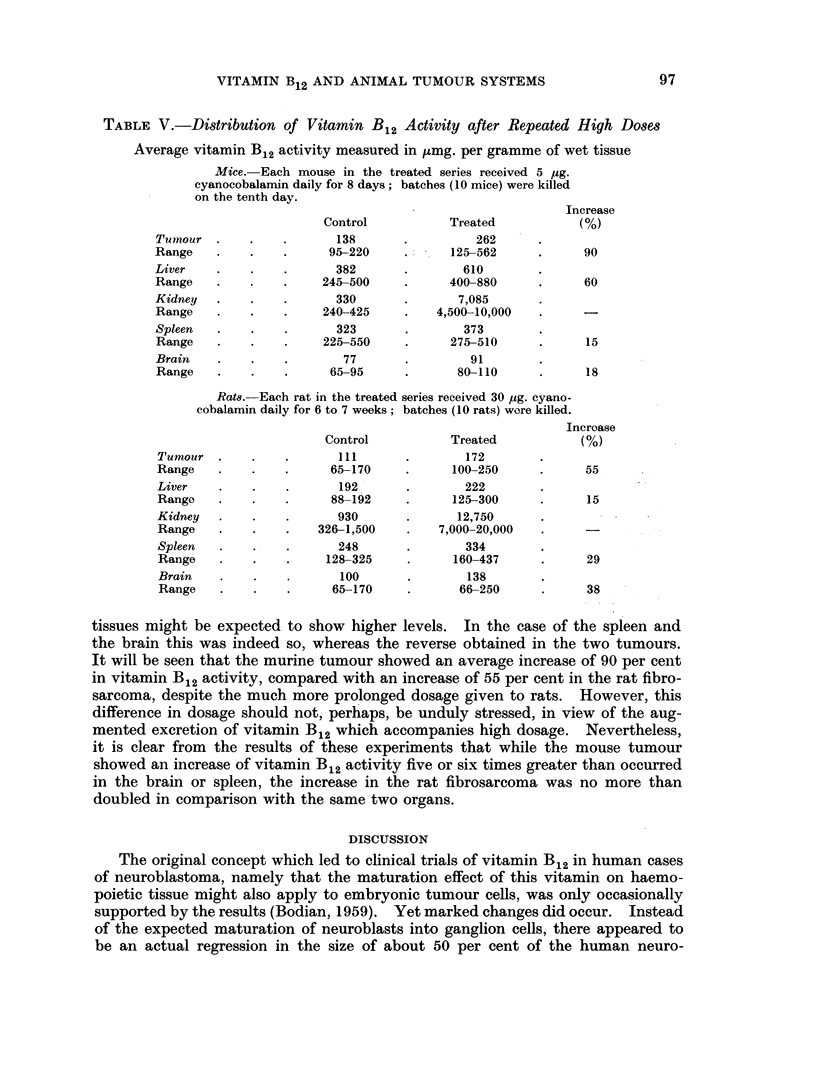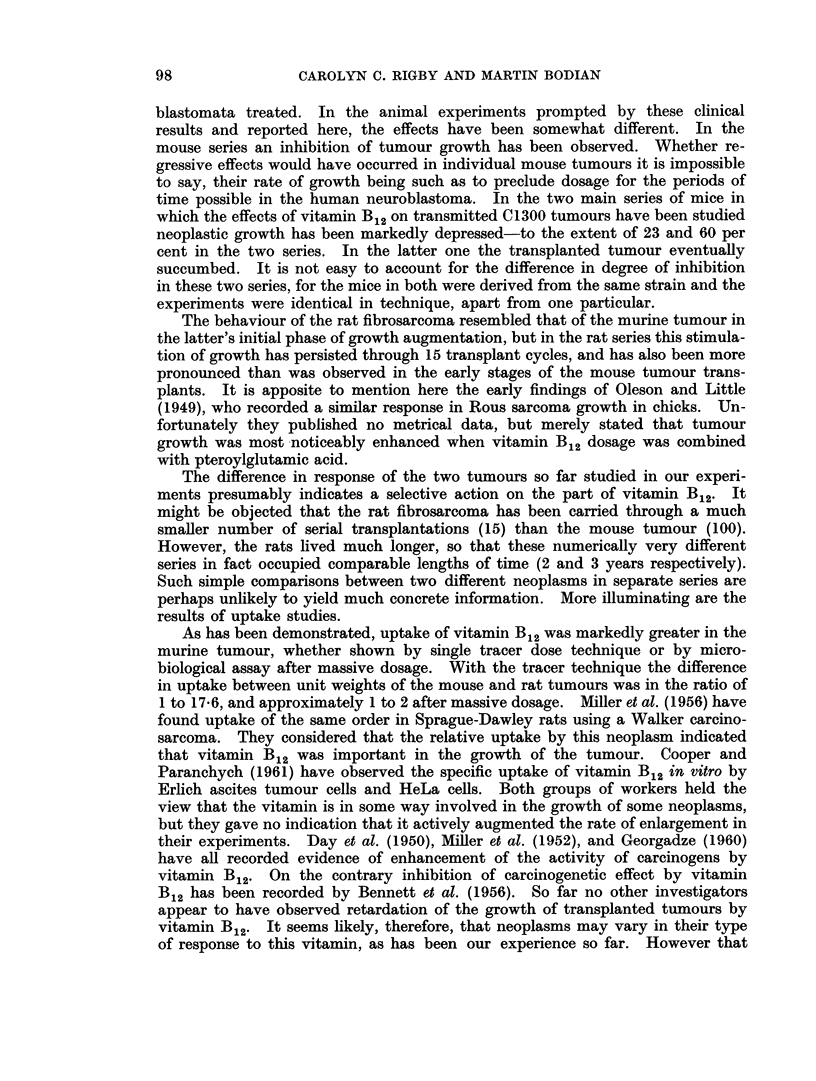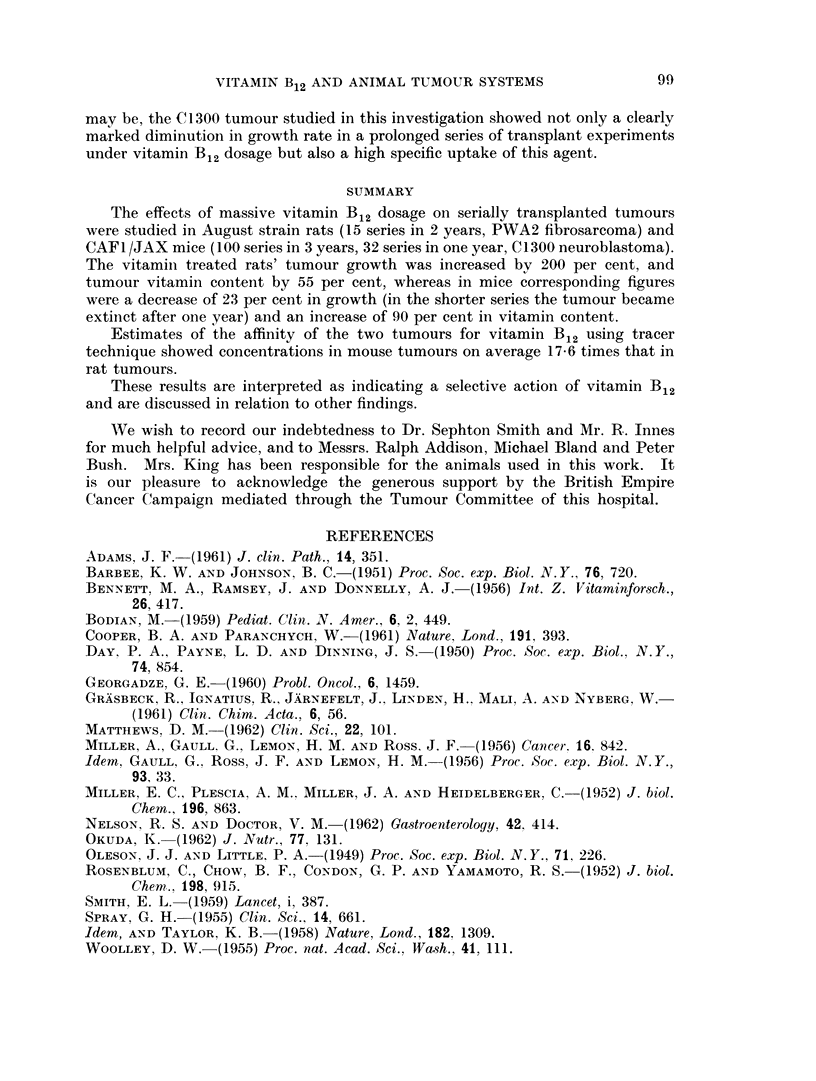# Experimental Study of the Relationship between Vitamin B_12_ and two Animal Tumour Systems

**DOI:** 10.1038/bjc.1963.14

**Published:** 1963-03

**Authors:** Carolyn C. Rigby, Martin Bodian


### EXPERIMENTAL STUDY OF THE RELATIONSHIP BETWEEN VITAMIN $B_{12}$ AND TWO ANIMAL TUMOUR SYSTEMS

CAROLYN C. RIGBY AND MARTIN BODIAN

*From The Department of Morbid Anatomy, The Hospital for Sick Children, Great Ormond Street, London*

Received for publication January 18, 1963

THE encouraging results of massive vitamin $B_{12}$ dosage in treating neuroblastoma in children (Bodian, 1959) suggested a parallel investigation of the effects of this vitamin on tumours occurring in experimental animals. Initial attempts to implant tissue from human neuroblastomata were unsuccessful, and therefore a spontaneous retroperitoneal tumour of mice was obtained (C1300, Roscoe B. Jackson Memorial Laboratory, Bar Harbour, Maine). This was originally considered to be a neuroblastoma, and although it has been shown histologically to be an undifferentiated sarcoma, it was decided to test the effects of vitamin $B_{12}$ upon it, together with a fibrosarcoma of rats (PWA2) to broaden the scope of the study.

If vitamin $B_{12}$ did affect tumour tissue, it would be reasonable to expect a relatively high uptake of the vitamin by such cells. The distribution of vitamin $B_{12}$ in neoplastic and other tissues has therefore been observed in mice and rats bearing tumour transplants. As far as can be ascertained the only workers who have reported studies of the relationship of vitamin $B_{12}$ to tumour growth are Oleson and Little (1949), Day, Payne and Dinning (1950), Miller *et al.* (1952), Woolley (1955), Bennett, Ramsey and Donnelly (1956), Miller *et al.* (1956), Georgadze (1960), Cooper and Paranchych (1961), and Nelson and Doctor (1962).

### MATERIALS AND METHODS

The mice used in this investigation were of CAF1/JAX strain, obtained from the Jackson Memorial Laboratory, which was also the source of the tumour with which they were inoculated. The rats were of the August strain and were supplied by the Chester Beatty Research Institute together with the fibrosarcoma (PWA2) used for transplantation in these animals. Mice were fed on standard cubes (Diet 41) and rats similarly (Diet 86).

The basic method pursued has been to take tissue from a tumour in one animal and to inoculate this into a number of other hosts, allowing these transplants to grow and repeating the transplantation to further hosts at regular intervals. In each successive group of animals, all affected with the tumour, half received vitamin $B_{12}$ and half acted as untreated controls.

In studying the behaviour of the murine tumour under such conditions, mature mice about 20 grammes in weight were inoculated in batches of 20 (all of the same sex—male or female—in each batch) at a single session. The inoculant was prepared by dissecting out a tumour from an affected animal, placing this in an aqueous solution of penicillin and streptomycin, and removing from it 10 approximately equal samples for subcutaneous injection through a 14 S.W.G. Bashford needle into separate mice. It must be emphasised at this point that the tumours transplanted through control and vitamin-treated series of animals were kept strictly separate. Control mice thus always received tumour transplants from a control animal in the previous batch, and similarly in the case of treated animals. This segregation was essential because the tumour used displayed such a rapid rate of growth in mice that it was impracticable to keep the affected individuals alive and under treatment for more than 10 days. Thus the long-term behaviour of treated and untreated tumour tissues could only be compared by carrying them separately through repeated series of control and vitamin-treated hosts.

On the day after transplantation mice in the vitamin-treated series received their first daily dose, 5 $\mu$g. of cyanocobalamin, given subcutaneously. Eight such doses were administered, a total of 40 $\mu$g. to each animal. On the tenth day the whole batch of 10 treated and 10 untreated controls were killed and dissected. It was almost always comparatively easy to dissect the tumours, and their individual weights were noted. Control and vitamin-treated series have now been carried in parallel through 100 transplant cycles, spread over a period of nearly 3 years.

The rat fibrosarcoma has been studied in a similar manner, in parallel series of equal numbers of control and treated animals in each transplant cycle. Mature rats of about 200 grammes weight and of the same sex in each batch were inoculated with PWA2 fibrosarcoma by the same technique as used in mice, using a 6 S.W.G. needle. Because of the size of the latter, which required a skin incision, and of the greater difficulty of handling the rats, intraperitoneal anaesthesia was adopted. Initially, control and treated batches numbered 7 each in every transplant cycle, but this number has been gradually increased to 10 in succeeding cycles as stocks of rats improved. As in the case of mice the length of the transplant cycle was dictated by the rate of growth of the tumour, which proved much slower in the rat fibrosarcoma. It was found possible to keep rats alive for 5 to 7 weeks, during which period the treated animals received daily individual doses of 30 $\mu$g. of cyanocobalamin. At the end of each transplant cycle normal control and treated rats were killed and dissected, like the mice, and the same data recorded. Transplants have now been passed through 15 cycles, during a period of about 2 years.

*Radioactive vitamin technique*

Radioactive vitamin $B_{12}$ was used as the $^{58}$Co isotope for injection in tracer doses into mice and rats to estimate distribution of the vitamin in various tissues, including tumours. It is well to recognise that radioactivity is assessed in such experiments, and that results give a measure of the amounts of cobalt isotope present, not of vitamin $B_{12}$. If these should be dissociated in a particular tissue, results would be fallacious. Barbee and Johnson (1951) have shown that such dissociation occurs in the gut after oral dosage in rats ; but they reported that chromatograms suggested that the $^{60}$Co isotope was stored as vitamin $B_{12}$ in the kidney. Rosenblum *et al.* (1952) demonstrated that the radioactivity in urine after parenteral doses of $^{60}$Co $B_{12}$ was due to intact radiovitamin, and Adams (1961) has recorded similar findings in man. Gräsbeck *et al.* (1961) concluded from their experiments in rats that after parenteral doses levels of activity in kidney, liver, spleen, and a number of other tissues could be taken as a measure of the take-up of radiovitamin.

Supplies of $^{58}$Co $B_{12}$ were obtained from the Radiochemical Centre, Amersham, having a specific activity of about 1 $\mu$c/1 $\mu$g. Since it has been shown that radiovitamin $B_{12}$ is unstable in solution (Smith, 1959), only fresh aqueous solutions were used. Dosage was adjusted between the dictates of counting technique and physiological levels ; suitable doses were found to be 0·075 $\mu$g. for mice and 0·2 $\mu$g. for rats. Animals received a single subcutaneous dose and were killed in batches of 10 at intervals of 4, 24, 48, 72 and 96 hours thereafter. Each batch of 10 consisted of 5 normal and 5 tumour-inoculated animals.

The mouse neuroblastoma is a rapidly growing tumour and tends to ulcerate early. It was therefore necessary to select animals for tracer injections at the right period of tumour growth. This was most conveniently achieved by maintaining an adequate stock of transplanted animals, from which batches of 5 could be taken for injection at the appropriate stage of tumour size. This difficulty did not affect experiments on rats, because of the slower rate of growth of the PWA2 fibrosarcoma.

Batches of 10 animals (5 tumour-transplanted, 5 normal controls) were taken at the intervals noted above, and were anaesthetized, heparinized, and exsanguinated. Individuals in each batch were killed in the same order in which they had been injected to keep survival intervals as uniform as possible. Each mouse or rat was then carefully dissected to remove the tumour (except in the controls), liver, kidneys, spleen, and brain (rats only), and a sample of blood was also collected.

The radioactivity of each specimen from every animal was measured separately in a well-type scintillation counter using a thallium-activated iodide crystal, each organ giving a count of well above 10 times the background activity. Total radioactivity injected into each animal was calculated from a count on a measured specimen of the solution used.

*Microbiological assay*

For microbiological assay the tumours, livers, kidneys, spleens, and brains were dissected from mice and rats and stored at $-10°$ C., the animals being taken from the main experimental series. From each transplant group assayed the above organs were separately pooled, so that there were 5 pools from the vitamin-treated animals and 5 from the batch controls. Each tissue pool was chopped up and extracted in a buffered (pH 4·8) cyanide-activated papain solution in the ratio of 1 to 20 by volume. The vitamin $B_{12}$ activity of diluted samples of the supernatant extract was assessed by the growth of *Lactobacillus leichmannii* 313 (ATCC7830), using Dano-$B_{12}$ assay medium, after the method described by Spray (1955) and Matthews (1962).

Spray and Taylor (1958) have shown that, using Lactobacillus, 50 per cent of vitamin $B_{12}$ activity is alkali-stable in rat serum, though the fraction in liver tissue was less than 3 per cent. In agreement with this our assays demonstrated a similar low alkali-stable activity in liver and also in the other tissues involved in this study. This fraction was therefore ignored.

Vitamin $B_{12}$ activity of the sample tissue extracts was estimated by photoelectric comparison of the density of bacillus growth in these and in standard solutions of the vitamin, after incubation for 20 hours at 37° C. in a uniformly illuminated waterbath. It was found that the stock of test organism could be most conveniently maintained in a gelatin base medium, which produced dispersed cultures easier for sampling.

### RESULTS

### 1. *Effects of Massive Dosage of Vitamin $B_{12}$*

*(a) C1300 mouse neuroblastoma in CAF1/JAX strain mice*

The effects of prolonged massive dosage of vitamin $B_{12}$ on the murine tumour have been followed continuously by serial transplantation since August, 1959. To date 100 successive batches have been carried through, together with equal numbers of animals which were tumour-inoculated but received no vitamin, thus acting as controls. Initially the mice receiving vitamin $B_{12}$ showed an increase in average tumour weight when compared with the control animals ; but an opposite effect soon supervened. This diminution in growth of transplants has persisted in vitamin-treated mice ; and while there have been slight vagaries from this trend in individual mice, the overall pattern in the last 88 transplant cycles has shown an average diminution of 23 per cent.

A second and identical tumour transplant series was started later than the above experiment, using a fresh sample of the same tumour. It was carried on in parallel through a further 32 transplantations. At this juncture, about a year after the commencement of this series, the tumour became extinct in the treated mice. At 10 months, shortly before the tumour became extinct, it showed a growth diminution of 60 per cent in the vitamin-treated animals, compared with the controls.

*(b) PWA2 fibrosarcoma in August strain rats*

Long term experiments similar to the above have also been carried out in rats to study the effects of massive vitamin $B_{12}$ dosage on a different tumour, the PWA2 fibrosarcoma. Because of its slower growth rats must be kept alive much longer, and only 15 successive transplants have been carried through in a period of 2 years. Some variation in the behaviour of the tumours in the vitamin-treated rats has occurred, as is also true of the control animals ; but in the treated animals, taken as a whole, there has been on average a threefold increase in the weight of tumours, compared with controls.

### 2. *Relative Uptake of Vitamin $B_{12}$*

*(a) Distribution of radioactivity after single small injections of cobalt-labelled vitamin $B_{12}$ ($^{58}Co$ $B_{12}$)*

The distribution of radioactivity in certain tissues, including tumours, after tracer doses of vitamin $B_{12}$ is shown in Tables I to IV. Both mice and rats were studied in groups of 5, with an equal number of normal animals. In both series of experiments 5 groups of animals (5 tumour-bearing and 5 normals in each) were killed at intervals of 4 to 96 hours, as the tables show. As will be evident, the distribution of radioactivity in tumours, liver, spleen, brain, and blood has been expressed in both relative and absolute terms. In Tables I and II the results are shown as percentages of the total injected dose, compared on the basis

TABLE I.—*Distribution of Radioactivity after Tracer Doses of* $^{58}Co$ *Vitamin* $B_{12}$

Average percentage of injected dose present in 1 gramme of wet tissue

| | Time in hours: 4 | 24 | 48 | 72 | 96 |
|---|---|---|---|---|---|
| | Tumour-bearing series of mice | | | | |
| *Tumour* . . . | 3·6 | 4·4 | 8·3 | 2·9 | 9·0 |
| Range . . . | 2·1–5·2 | 2·5–9·0 | 4·7–10·1 | 2·3–3·6 | 3·4–15·5 |
| *Liver* . . . | 12·2 | 12·9 | 14·5 | 17·6 | 15·4 |
| Range . . . | 9·9–14·3 | 9·8–15·4 | 10·9–17·4 | 14·7–21·6 | 11·8–17·3 |
| *Kidneys* . . . | 10·1 | 7·5 | 4·7 | 4·8 | 4·1 |
| Range . . . | 7·0–15·3 | 6·2–10·0 | 3·9–5·2 | 4·0–5·5 | 2·8–5·7 |
| *Spleen* . . . | 0·6 | 0·7 | 0·6 | 0·8 | 0·7 |
| Range . . . | 0·4–0·9 | 0·6–0·7 | 0·5–0·8 | 0·6–1·3 | 0·5–0·9 |
| *Blood* (total) . . | 5·6 | 5·3 | 3·5 | 2·6 | 2·2 |
| Range . . . | 3·3–9·4 | 4·2–6·2 | 1·6–5·7 | 2·0–3·6 | 1·4–3·4 |
| | Control series of mice | | | | |
| *Liver* . . . | 12·9 | 15·6 | 15·3 | 17·0 | 19·2 |
| Range . . . | 12·0–14·1 | 14·8–16·1 | 13·2–17·4 | 15·4–18·8 | 17·3–21·2 |
| *Kidneys* . . . | 11·0 | 6·3 | 6·8 | 5·5 | 6·3 |
| Range . . . | 8·2–17·2 | 6·1–6·4 | 5·3–11·7 | 4·1–6·7 | 6·1–6·5 |
| *Spleen* . . . | 0·6 | 0·8 | 0·7 | 0·8 | 0·6 |
| Range . . . | 0·5–0·8 | 0·6–1·2 | 0·7–0·8 | 0·6–1·3 | 0·1–1·0 |
| *Blood* (total) . . | 9·2 | 5·6 | 2·9 | 3·1 | 1·9 |
| Range . . . | 7·7–13·0 | 4·0–8·9 | 2·4–3·2 | 1·2–4·4 | 1·2–2·3 |

Each mouse received 0·075 μg.

TABLE II.—*Distribution of Radioactivity after Tracer Doses of* $^{58}Co$ *Vitamin* $B_{12}$

Average percentage of injected dose present in whole organs

| | Time in hours: 4 | 24 | 48 | 72 | 96 |
|---|---|---|---|---|---|
| | Tumour-bearing series of rats | | | | |
| *Tumour* . . . | 1·20 | 0·26 | 0·83 | 0·94 | 0·60 |
| Range . . . | 0·6–1·8 | 0·1–0·4 | 0·005–3·1 | 0·6–1·4 | 0·3–0·7 |
| *Liver* . . . | 4·18 | 3·26 | 4·16 | 4·16 | 4·44 |
| Range . . . | 4·0–4·3 | 2·7–3·6 | 3·6–4·8 | 3·9–4·8 | 3·7–4·8 |
| *Kidneys* . . . | 16·88 | 18·74 | 17·66 | 17·88 | 17·74 |
| Range . . . | 16·1–18·4 | 16·0–20·9 | 15·6–19·8 | 15·9–20·0 | 14·9–20·2 |
| *Spleen* . . . | 1·50 | 0·76 | 0·74 | 0·68 | 0·80 |
| Range . . . | 1·3–1·7 | 0·7–0·8 | 0·6–0·8 | 0·6–0·8 | 0·6–1·0 |
| *Blood* (total) . . | 1·56 | 0·50 | 0·44 | 0·32 | 0·44 |
| Range . . . | 1·0–2·0 | 0·4–0·6 | 0·3–0·8 | 0·2–0·5 | 0·1–0 6 |
| | Control series of rats | | | | |
| *Liver* . . . | 3·92 | 4·00 | 4·06 | 5·10 | 5·38 |
| Range . . . | 3·8–4·3 | 3·6–4·2 | 2·6–4·6 | 4·8–5·6 | 5·1–5·6 |
| *Kidneys* . . . | 15·24 | 22·12 | 18·84 | 17·86 | 21·94 |
| Range . . . | 14·2–16·6 | 19·6–25·2 | 10·8–23·6 | 14·7–19·3 | 19·3–25·5 |
| *Spleen* . . . | 1·38 | 0·84 | 0·66 | 0·90 | 0·54 |
| Range . . . | 1·2–1·6 | 0·6–1·3 | 0·4–1·0 | 0·7–1·1 | 0·4–0·8 |
| *Blood* (total) . . | 0·96 | 0·64 | 0·54 | 0·60 | 0·48 |
| Range . . . | 0·8–1·1 | 0·2–0·8 | 0·3–0·8 | 0·3–1·0 | 0·3–0·6 |

Each rat received 0·2 μg. Batches of five animals killed at the above intervals of time.

TABLE III.—*Distribution of Radioactivity after Tracer Doses of $^{58}Co$ Vitamin $B_{12}$*

Average percentage of injected dose present in 1 gramme of wet tissue

| | Time in hours | | | | |
|---|---|---|---|---|---|
| | 4 | 24 | 48 | 72 | 96 |
| | Tumour-bearing series of mice | | | | |
| *Tumour* . . . | 10·7 | 17·0 | 14·6 | 13·2 | 12·8 |
| Range . . . | 7·4–15·3 | 14·1–21·4 | 10·9–18·1 | 7·3–18·1 | 10·0–15·9 |
| *Liver* . . . | 12·0 | 13·0 | 15·7 | 15·8 | 14·8 |
| Range . . . | 10·2–14·6 | 10·6–15·4 | 11·4–23·2 | 11·8–19·6 | 10·8–17·9 |
| *Kidneys* . . . | 39·7 | 31·7 | 19·6 | 18·6 | 17·0 |
| Range . . . | 29·0–58·6 | 24·2–45·6 | 17·1–23·5 | 15·0–23·1 | 10·4–23·0 |
| *Spleen* . . . | 4·7 | 5·7 | 5·4 | 6·4 | 5·1 |
| Range . . . | 3·3–5·7 | 5·1–6·3 | 4·8–6·2 | 5·2–9·8 | 4·2–6·5 |
| *Blood* (total) . . | 5·6 | 5·3 | 3·5 | 2·6 | 2·2 |
| Range . . . | 3·3–9·4 | 4·2–6·2 | 1·6–5·7 | 2·0–3·6 | 1·4–3·4 |
| | Control series of mice | | | | |
| *Liver* . . . | 13·1 | 16·0 | 15·0 | 18·8 | 14·9 |
| Range . . . | 11·7–14·4 | 14·2–17·7 | 12·6–16·8 | 16·7–20·9 | 14·5–15·6 |
| *Kidneys* . . . | 53·4 | 26·6 | 26·5 | 24·3 | 16·0 |
| Range . . . | 34·1–101 | 22·1–33·6 | 17·6–50·7 | 20·0–28·1 | 14·7–18·0 |
| *Spleen* . . . | 4·6 | 6·1 | 5·4 | 5·8 | 4·1 |
| Range . . . | 4·0–5·0 | 4·9–7·1 | 4·3–6·6 | 5·0–7·2 | 1·0–7·2 |
| *Blood* (total) . . | | | | | |
| Range . . . | 7·7–13·0 | 4·0–8·9 | 2·4–3·2 | 1·2–4·4 | 1·2–2·3 |

Each mouse received 0·075 μg. Batches of five animals killed at varying intervals of time.

TABLE IV.—*Distribution of Radioactivity after Tracer Doses of $^{58}Co$ Vitamin $B_{12}$*

Average percentage of injected dose present in 1 gramme of wet tissue

| | Time in hours | | | | |
|---|---|---|---|---|---|
| | 4 | 24 | 48 | 72 | 96 |
| | Tumour-bearing series of rats | | | | |
| *Tumour* . . . | 0·94 | 0·76 | 0·82 | 0·60 | 0·48 |
| Range . . . | 0·7–1·1 | 0·6–0·9 | 0·2–2·3 | 0·4–1·0 | 0·3–0·6 |
| *Liver* . . . | 0·34 | 0·42 | 0·54 | 0·48 | 0·38 |
| Range . . . | 0·3–0·4 | 0·3–0·6 | 0·4–0·7 | 0·4–0·5 | 0·3–0·4 |
| *Kidneys* . . . | 7·9 | 9·24 | 8·48 | 8·42 | 8·32 |
| Range . . . | 6·1–9·3 | 7·2–10·2 | 7·1–10·2 | 7·4–11·7 | 7·3–9·3 |
| *Spleen* . . . | 1·48 | 0·96 | 0·84 | 0·76 | 0·66 |
| Range . . . | 1·4–1·6 | 0·9–1·1 | 0·7–0·9 | 0·7–0·9 | 0·6–0·7 |
| *Blood* (total) . . | 1·56 | 0·50 | 0·44 | 0·32 | 0·44 |
| Range . . . | 1·0–2·0 | 0·4–0·6 | 0·3–0·8 | 0·2–0·5 | 0·1–0·6 |
| | Control series of rats | | | | |
| *Liver* . . . | 0·4 | 0·36 | 0·46 | 0·52 | 0·54 |
| Range . . . | . . | 0·3–0·5 | 0·4–0·6 | 0·5–0·6 | 0·5–0·6 |
| *Kidneys* . . . | 7·82 | 11·34 | 8·68 | 7·9 | 10·2 |
| Range . . . | 7·3–8·5 | 8·9–14·7 | 6·1–11·3 | 6·2–8·8 | 9·3–11·0 |
| *Spleen* . . . | 1·66 | 0·98 | 0·80 | 0·68 | 0·76 |
| Range . . . | 1·5–1·8 | 0·6–1·3 | 0·6–1·1 | 0·5–0·8 | 0·6–0·9 |
| *Blood* (total) . . | 0·96 | 0·64 | 0·54 | 0·60 | 0·48 |
| Range . . . | 0·8–1·1 | 0·2–0·8 | 0·3–0·8 | 0·3–1·0 | 0·3–0·6 |

Each rat received 0·2 μg. Batches of five animals killed at above intervals of time.

of counts in unit time ; they thus express the amounts found in whole organs. Results set out in this way do not permit direct comparison of the concentrations in individual tissues. This comparison can only be made by calculating the percentages of injected counts taken up by unit mass of the different tissues, as has been done for Tables III and IV.

Regarding the organs selected for examination, the liver is the storage organ in the mouse (Miller *et al.*, 1956) and the kidney in the rat (Okuda, 1962). Since the kidney is an excretory organ with respect to vitamin $B_{12}$ in both species, high concentrations are to be expected in both these organs, in experiments on a short time basis. Originally a considerable variety of other organs was examined, and the spleen was chosen from these as a convenient representative of those showing relatively low radioactivity. Blood concentration was a necessary consideration because it was not practicable to assess the radioactivity of an organ separately from its blood content.

As will be at once apparent from the tables of results the murine tumour showed concentrations much higher than were found in the spleen. Tumour radioactivity reached a maximum in the first 24 hours after injection, showing thereafter a gentle fall. This descent might be due to excretion of the vitamin ; but since over the period covered by the experiments the blood concentrations displayed only a slow fall, it is more likely that the diminution in tumour radioactivity reflected its rapid growth—perhaps in conjunction with a falling blood level. Despite this, the tumours showed, at 96 hours after tracer injection, levels of radioactivity not far short of those in the liver.

In contrast to the mouse neoplasm, the fibrosarcoma investigated in rats showed no outstanding concentrations in comparison with other tissues. In comparing the two series of experiments, however, two difficulties must be pointed out. Firstly, the rats received a proportionately smaller dose of radiovitamin (approximately one quarter of the dose given to mice). Secondly, the liver does not act as a storage organ in the rat. For both these resons the levels of radioactivity in rat livers were much lower than in mice. Since the kidney is both a storage and an excretory organ in the rat, it also affords no simple comparison. It will nevertheless be noted that in rats the tumour concentrations of vitamin $B_{12}$ activity were no more than levels found in their spleens.

(*b*) *Distributions of vitamin $B_{12}$ activity after massive and prolonged dosage as estimated by microbiological assay*

In order to examine the distribution of vitamin $B_{12}$ activity following high and prolonged dosage, groups of rats and mice were taken from the main experimental series of the investigation. Five batches of rats and 10 of mice were studied, together with equal numbers of untreated animals as controls. It is to be noted that in these series the control animals had received tumour transplants.

The results of these experiments are shown in Table V. The kidneys, of course, show very large increases in vitamin $B_{12}$ activity in both species, indicative of excretion. Liver levels were much raised in mice but not in rats, corroborating existing views of this organ's different roles in the two animals in relation to vitamin $B_{12}$ storage. This difference is all the more noteworthy in consideration of the fact that the rats lived four to five times as long as the mice. Since daily vitamin dosage was in these experiments approximately proportionate to body weight, the rats received several times more vitamin than the mice, and their

TABLE V.—*Distribution of Vitamin $B_{12}$ Activity after Repeated High Doses*

Average vitamin $B_{12}$ activity measured in $\mu$mg. per gramme of wet tissue

*Mice.*—Each mouse in the treated series received 5 $\mu$g. cyanocobalamin daily for 8 days ; batches (10 mice) were killed on the tenth day.

| | Control | Treated | Increase (%) |
|---|---|---|---|
| *Tumour* | 138 | 262 | |
| Range | 95–220 | 125–562 | 90 |
| *Liver* | 382 | 610 | |
| Range | 245–500 | 400–880 | 60 |
| *Kidney* | 330 | 7,085 | |
| Range | 240–425 | 4,500–10,000 | — |
| *Spleen* | 323 | 373 | |
| Range | 225–550 | 275–510 | 15 |
| *Brain* | 77 | 91 | |
| Range | 65–95 | 80–110 | 18 |

*Rats.*—Each rat in the treated series received 30 $\mu$g. cyanocobalamin daily for 6 to 7 weeks ; batches (10 rats) were killed.

| | Control | Treated | Increase (%) |
|---|---|---|---|
| *Tumour* | 111 | 172 | |
| Range | 65–170 | 100–250 | 55 |
| *Liver* | 192 | 222 | |
| Range | 88–192 | 125–300 | 15 |
| *Kidney* | 930 | 12,750 | |
| Range | 326–1,500 | 7,000–20,000 | — |
| *Spleen* | 248 | 334 | |
| Range | 128–325 | 160–437 | 29 |
| *Brain* | 100 | 138 | |
| Range | 65–170 | 66–250 | 38 |

tissues might be expected to show higher levels. In the case of the spleen and the brain this was indeed so, whereas the reverse obtained in the two tumours. It will be seen that the murine tumour showed an average increase of 90 per cent in vitamin $B_{12}$ activity, compared with an increase of 55 per cent in the rat fibrosarcoma, despite the much more prolonged dosage given to rats. However, this difference in dosage should not, perhaps, be unduly stressed, in view of the augmented excretion of vitamin $B_{12}$ which accompanies high dosage. Nevertheless, it is clear from the results of these experiments that while the mouse tumour showed an increase of vitamin $B_{12}$ activity five or six times greater than occurred in the brain or spleen, the increase in the rat fibrosarcoma was no more than doubled in comparison with the same two organs.

### DISCUSSION

The original concept which led to clinical trials of vitamin $B_{12}$ in human cases of neuroblastoma, namely that the maturation effect of this vitamin on haemopoietic tissue might also apply to embryonic tumour cells, was only occasionally supported by the results (Bodian, 1959). Yet marked changes did occur. Instead of the expected maturation of neuroblasts into ganglion cells, there appeared to be an actual regression in the size of about 50 per cent of the human neuroblastomata treated. In the animal experiments prompted by these clinical results and reported here, the effects have been somewhat different. In the mouse series an inhibition of tumour growth has been observed. Whether regressive effects would have occurred in individual mouse tumours it is impossible to say, their rate of growth being such as to preclude dosage for the periods of time possible in the human neuroblastoma. In the two main series of mice in which the effects of vitamin $B_{12}$ on transmitted C1300 tumours have been studied neoplastic growth has been markedly depressed—to the extent of 23 and 60 per cent in the two series. In the latter one the transplanted tumour eventually succumbed. It is not easy to account for the difference in degree of inhibition in these two series, for the mice in both were derived from the same strain and the experiments were identical in technique, apart from one particular.

The behaviour of the rat fibrosarcoma resembled that of the murine tumour in the latter's initial phase of growth augmentation, but in the rat series this stimulation of growth has persisted through 15 transplant cycles, and has also been more pronounced than was observed in the early stages of the mouse tumour transplants. It is apposite to mention here the early findings of Oleson and Little (1949), who recorded a similar response in Rous sarcoma growth in chicks. Unfortunately they published no metrical data, but merely stated that tumour growth was most noticeably enhanced when vitamin $B_{12}$ dosage was combined with pteroylglutamic acid.

The difference in response of the two tumours so far studied in our experiments presumably indicates a selective action on the part of vitamin $B_{12}$. It might be objected that the rat fibrosarcoma has been carried through a much smaller number of serial transplantations (15) than the mouse tumour (100). However, the rats lived much longer, so that these numerically very different series in fact occupied comparable lengths of time (2 and 3 years respectively). Such simple comparisons between two different neoplasms in separate series are perhaps unlikely to yield much concrete information. More illuminating are the results of uptake studies.

As has been demonstrated, uptake of vitamin $B_{12}$ was markedly greater in the murine tumour, whether shown by single tracer dose technique or by microbiological assay after massive dosage. With the tracer technique the difference in uptake between unit weights of the mouse and rat tumours was in the ratio of 1 to 17·6, and approximately 1 to 2 after massive dosage. Miller *et al.* (1956) have found uptake of the same order in Sprague-Dawley rats using a Walker carcinosarcoma. They considered that the relative uptake by this neoplasm indicated that vitamin $B_{12}$ was important in the growth of the tumour. Cooper and Paranchych (1961) have observed the specific uptake of vitamin $B_{12}$ *in vitro* by Erlich ascites tumour cells and HeLa cells. Both groups of workers held the view that the vitamin is in some way involved in the growth of some neoplasms, but they gave no indication that it actively augmented the rate of enlargement in their experiments. Day *et al.* (1950), Miller *et al.* (1952), and Georgadze (1960) have all recorded evidence of enhancement of the activity of carcinogens by vitamin $B_{12}$. On the contrary inhibition of carcinogenetic effect by vitamin $B_{12}$ has been recorded by Bennett *et al.* (1956). So far no other investigators appear to have observed retardation of the growth of transplanted tumours by vitamin $B_{12}$. It seems likely, therefore, that neoplasms may vary in their type of response to this vitamin, as has been our experience so far. However that may be, the C1300 tumour studied in this investigation showed not only a clearly marked diminution in growth rate in a prolonged series of transplant experiments under vitamin $B_{12}$ dosage but also a high specific uptake of this agent.

SUMMARY

The effects of massive vitamin $B_{12}$ dosage on serially transplanted tumours were studied in August strain rats (15 series in 2 years, PWA2 fibrosarcoma) and CAF1/JAX mice (100 series in 3 years, 32 series in one year, C1300 neuroblastoma). The vitamin treated rats' tumour growth was increased by 200 per cent, and tumour vitamin content by 55 per cent, whereas in mice corresponding figures were a decrease of 23 per cent in growth (in the shorter series the tumour became extinct after one year) and an increase of 90 per cent in vitamin content.

Estimates of the affinity of the two tumours for vitamin $B_{12}$ using tracer technique showed concentrations in mouse tumours on average 17·6 times that in rat tumours.

These results are interpreted as indicating a selective action of vitamin $B_{12}$ and are discussed in relation to other findings.

We wish to record our indebtedness to Dr. Sephton Smith and Mr. R. Innes for much helpful advice, and to Messrs. Ralph Addison, Michael Bland and Peter Bush. Mrs. King has been responsible for the animals used in this work. It is our pleasure to acknowledge the generous support by the British Empire Cancer Campaign mediated through the Tumour Committee of this hospital.

### REFERENCES

Adams, J. F.—(1961) *J. clin. Path.*, **14**, 351.

Barbee, K. W. and Johnson, B. C.—(1951) *Proc. Soc. exp. Biol. N.Y.*, **76**, 720.

Bennett, M. A., Ramsey, J. and Donnelly, A. J.—(1956) *Int. Z. Vitaminforsch.*, **26**, 417.

Bodian, M.—(1959) *Pediat. Clin. N. Amer.*, **6**, 2, 449.

Cooper, B. A. and Paranchych, W.—(1961) *Nature, Lond.*, **191**, 393.

Day, P. A., Payne, L. D. and Dinning, J. S.—(1950) *Proc. Soc. exp. Biol., N.Y.*, **74**, 854.

Georgadze, G. E.—(1960) *Probl. Oncol.*, **6**, 1459.

Gräsbeck, R., Ignatius, R., Järnefelt, J., Linden, H., Mali, A. and Nyberg, W.—(1961) *Clin. Chim. Acta.*, **6**, 56.

Matthews, D. M.—(1962) *Clin. Sci.*, **22**, 101.

Miller, A., Gaull, G., Lemon, H. M. and Ross, J. F.—(1956) *Cancer*, **16**, 842.

*Idem*, Gaull, G., Ross, J. F. and Lemon, H. M.—(1956) *Proc. Soc. exp. Biol. N.Y.*, **93**, 33.

Miller, E. C., Plescia, A. M., Miller, J. A. and Heidelberger, C.—(1952) *J. biol. Chem.*, **196**, 863.

Nelson, R. S. and Doctor, V. M.—(1962) *Gastroenterology*, **42**, 414.

Okuda, K.—(1962) *J. Nutr.*, **77**, 131.

Oleson, J. J. and Little, P. A.—(1949) *Proc. Soc. exp. Biol. N.Y.*, **71**, 226.

Rosenblum, C., Chow, B. F., Condon, G. P. and Yamamoto, R. S.—(1952) *J. biol. Chem.*, **198**, 915.

Smith, E. L.—(1959) *Lancet*, i, 387.

Spray, G. H.—(1955) *Clin. Sci.*, **14**, 661.

*Idem*, and Taylor, K. B.—(1958) *Nature, Lond.*, **182**, 1309.

Woolley, D. W.—(1955) *Proc. nat. Acad. Sci., Wash.*, **41**, 111.